# Tailoring CuO/Polyaniline Nanocomposites for Optoelectronic Applications: Synthesis, Characterization, and Performance Analysis

**DOI:** 10.3390/polym17101423

**Published:** 2025-05-21

**Authors:** Fedda Alzoubi, Mahmoud Al-Gharram, Tariq AlZoubi, Hasan Al-Khateeb, Mohammed Al-Qadi, Osamah Abu Noqta, Ghaseb Makhadmeh, Omar Mouhtady, Mohannad Al-Hmoud, Jestin Mandumpal

**Affiliations:** 1 Physics Department, Jordan University of Science and Technology, Irbid 22110, Jordan; 2 Department of Physics, School of Electrical Engineering and Information Technology, German Jordanian University, Amman 11180, Jordan; 3 College of Engineering and Technology, American University of the Middle East, Egaila 54200, Kuwait; omar.mouhtady@aum.edu.kw (O.M.); jestin.mandumpal@aum.edu.kw (J.M.); 4 MEU Research Unit, Middle East University, Amman 11831, Jordan; 5 General Education Department, Skyline University College, Sharjah P.O. Box 1797, United Arab Emirates; 6 Department of Physics, College of Science, Imam Mohammad Ibn Saud Islamic University (IMSIU), Riyadh 13318, Saudi Arabia

**Keywords:** polyaniline, CuO NPs, nanocomposites, electrochemical polymerization, XRD, SEM

## Abstract

This research focuses on creating CuO/PANI nanocomposite films by electrodepositing copper oxide nanoparticles into a polyaniline matrix on ITO substrates. The CuO nanoparticle content was adjusted between 7% and 21%. These nanocomposites are promising for various applications, such as optoelectronic devices, gas sensors, electromagnetic interference shielding, and electrochromic devices. We utilized UV-Vis spectroscopy to examine the nanocomposites’ interaction with light, allowing us to ascertain their refractive indices and absorption coefficients. The Scherrer formula facilitated the determination of the average crystallite size, shedding light on the material’s internal structure. Tauc plots indicated a reduction in the energy-band gap from 3.36 eV to 3.12 eV as the concentration of CuO nanoparticles within the PANI matrix increased, accompanied by a rise in electrical conductivity. The incorporation of CuO nanoparticles into the polyaniline matrix appears to enhance the conjugation length of PANI chains, as evidenced by shifts in the quinoid and benzenoid ring vibrations in FTIR spectra. SEM analysis indicates that the nanocomposite films possess a relatively smooth and homogeneous surface. Additionally, FTIR and XRD analyses demonstrate an increasing degree of interaction between CuO nanoparticles and PANI chains with higher CuO concentrations. At lower concentrations, interactions were minimal. In contrast, at higher concentrations, more significant interactions were observed, which facilitated the stretching of polymer chains, improved molecular packing, and facilitated the formation of larger crystalline structures within the PANI matrix. The incorporation of CuO nanoparticles resulted in nanocomposites with electrical conductivities ranging from 1.2 to 17.0 S cm^−1^, which are favorable for optimum performance in optoelectronic devices. These results confirm that the nanocomposite films combine pronounced crystallinity, markedly enhanced electrical conductivity, and tunable band-gap energies, positioning them as versatile candidates for next-generation optoelectronic devices.

## 1. Introduction

Conducting polymer-metal oxide nanocomposites have emerged as a focal point in materials research. Recent studies have demonstrated that incorporating dielectric metal oxide nanoparticles into conducting polymer matrices can significantly enhance their optical and electronic properties [1,2,3,4,5,6,7,8,9]. Advanced nanocomposites are integral to the progression of modern technologies, encompassing electronics, optical sensors, electrochromic devices, and biomedical applications [10,11,12]. Copper (II) oxide (CuO) is valued for its cost-effectiveness, biocompatibility, chemical stability, high oxygen-storage capacity, and favorable electrochemical properties, making it a significant material in applications such as catalysis, energy storage, and optoelectronics [13]. Polyaniline (PANI) is a widely studied conducting polymer known for its tunable electrical properties, which can be optimized through doping and dedoping processes; nanostructured PANI exhibits enhanced performance due to its increased specific surface area. Several metal oxides, including V_2_O_5_ [14,15,16], TiO_2_ [17], CuO [18], and Nb_2_O_5_ [10,19,20], have been integrated into PANI matrices to improve material properties. Although studies have demonstrated that CuO/PANI composites exhibit enhanced electrical conductivity compared to their pure constituents [21], comprehensive analyses of their optical, structural, and electrical characteristics remain limited [18,22]. The intrinsic properties of CuO nanoparticles—such as a high surface area and strong adsorption capabilities—contribute significantly to the overall functionality of the nanocomposites [23,24,25,26]. Moreover, conducting polymers have attracted attention in diverse applications ranging from optical devices [3] to sensors [27], and their electrical conductivity may reach levels comparable to metals following proper doping [28,29].

This research focuses on fabricating nanocomposites by integrating CuO nanoparticles (NPs) into a PANI matrix. The strategy involves the electrochemical deposition of PANI with varying CuO NPs concentrations (7–21 wt%), which enables precise control over film morphology and dopant incorporation. Camphor sulfonic acid (CSA) is used for doping, enhancing the electrical conductivity of PANI while promoting uniform film formation through an environmentally friendly electrochemical polymerization process [30,31,32,33,34]. The resulting nanocomposites were comprehensively characterized by several analytical techniques: X-ray fluorescence (XRF) for elemental composition; 3D optical profilometry for surface topography and thickness; Fourier transform infrared (FTIR) spectroscopy for chemical structure; scanning electron microscopy (SEM) for surface morphology; X-ray diffraction (XRD) for crystalline structure; four-point probe measurements for electrical conductivity; and UV–Visible–NIR spectroscopy for optical properties.

In summary, our study demonstrates that integrating CuO nanoparticles into a PANI matrix markedly enhances the electro-optical properties of the resulting nanocomposites. Specifically, we observed significant improvements in electrical conductivity and surface morphology, attributable to the unique adsorption capacity and physical properties of CuO. Moreover, the use of electrochemical deposition allows for the synthesis of uniform, defect-free films with controlled thickness and dopant distribution. These findings not only validate the effectiveness of our fabrication strategy but also position CuO/PANI nanocomposites as promising materials for advanced applications in sensors, electrochromic devices, and energy-storage systems.

## 2. Experimental Techniques

### 2.1. Chemicals

Aniline, CSA, and CuO NPs (10 nm) were sourced from US-Nano, Sarasota, FL 34234, USA. A 2.5 cm × 3.0 cm ITO substrate (Ossila Ltd., S4 7WB, Sheffield, UK) functioned as the working electrode. Before deposition, the ITO substrates underwent thorough cleaning through sequential 10 min sonication in deionized water, ethanol, and acetone.

### 2.2. Films Electrodeposition

An electrolyte solution was prepared by dissolving aniline monomer (0.25 M) and CSA (0.4 M) in deionized water. CSA serves as both a protonating acid and a dopant in this process. This solution underwent chemical oxidative polymerization. Then, the aniline/CSA solution was divided into several equal portions, each mixed with a specific concentration of CuO NPs. Uniform dispersion of the CuO NPs within the aniline monomer solution was achieved by vortexing for 10 min. Three distinct CuO nanoparticle concentrations (7, 14, and 21 wt.%) were investigated. Electro-polymerization was performed in a single-compartment electrochemical cell at approximately 25 °C. The cell consisted of a stainless-steel counter electrode and the working electrode, separated by 1 cm using a Teflon spacer. Aniline monomers were oxidized electrochemically at an applied potential of 1.2 V vs. Ag/AgCl (3M KCl), serving as the reference electrode. During this process, aniline monomers are oxidized to form radical cations, which subsequently undergo coupling reactions at the electrode surface to form polyaniline chains. This method ensures precise control over film thickness, uniform incorporation of dopants, and is environmentally favorable compared to chemical polymerization techniques. These experimental conditions have now been clarified to ensure methodological accuracy and reproducibility. An optical profilometer revealed an average film thickness of approximately 2000 nm.

### 2.3. Electrochemical Oxidative Polymerization

PANI was synthesized through electrochemical polymerization. This process involves applying an electric potential or current between electrodes submerged in a solution containing the aniline monomer and a dopant [35]. The electrochemical approach facilitates the polymerization process, leading to the formation of polyaniline on the electrode surface.

**Figure A1:**
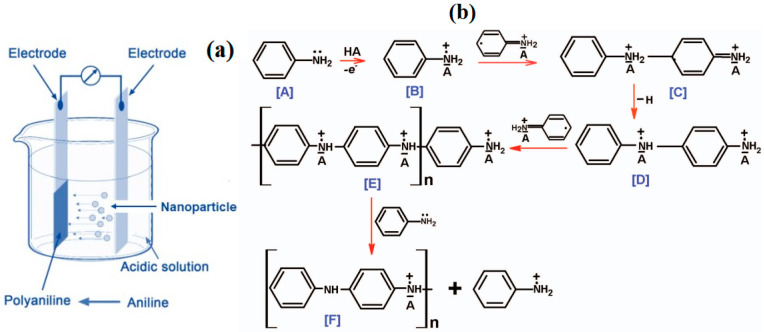
(**a**) Schematic illustration of the electrochemical cell used for PANI synthesis, (**b**) proposed mechanism for the electrochemical oxidative polymerization of aniline to form PANI [35].

Upon application of potential, aniline monomers are oxidized at the working electrode, losing electrons and forming radical cations. These radical cations then react with other aniline species in solution, leading to the formation of PANI chains. Continued electrochemical polymerization leads to the progressive deposition of the polymer on the electrode surface [34]. Figure A1a,b schematically illustrates the electrochemical polymerization of aniline.

### 2.4. Characterization Techniques

X-ray fluorescence (XRF) measurements were performed using a Rigaku NEXQC+ system, which employs a 50 kV, 4 W X-ray tube as the source. A helium purge was maintained, with the sample positioned at less than 1 cm from the detector. A silicon drift detector (SDD) with superior resolution was used to collect the emitted fluorescence signals. The surface characterization was performed using a Filmetrics Profilm 3D optical profilometer, Milpitas, CA, USA, which utilizes white light interferometry (WLI) to measure surface roughness and topography with high precision. The chemical composition and structure of the nanocomposites were investigated using FTIR spectroscopy (PerkinElmer, Waltham, MA, USA) in the range of 4000–400 cm^−1^. The optical properties of the samples were analyzed utilizing a Shimadzu-100 UV-Vis spectrophotometer ranging from 200 nm to 3300 nm, equipped with an integrating sphere, and it has Tungsten halogen visible and deuterium arc UV. This configuration allowed for the measurement of light transmitted through the film and substrate, effectively capturing the total light interaction with the sample. The surface morphology of the films was investigated using a Quanta FEG 450 SEM, Graz, Austria, operated at 20 kV. Crystal structure analysis was performed via XRD using a Shimadzu XRD-7000 diffractometer, Kyoto, Japan. The XRD patterns were acquired over a 2θ range of 5° to 70° with a step size of 0.02°. A Cu Kα X-ray source (λ = 1.5406 Å) was used, and the system was configured in a conventional Bragg–Brentano geometry to obtain high-resolution diffraction patterns. The current-voltage (*I*–*V*) characteristics were determined using a four-point probe technique.

## 3. Results and Discussion

### 3.1. XRF Characterization

**Figure 1:**
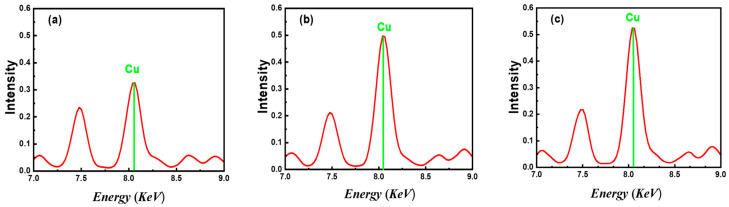
X-ray fluorescence (XRF) spectra of CuO/PANI nanocomposites with varying CuO NPs concentrations: (**a**) 7 wt%, (**b**) 14 wt%, and (**c**) 21 wt%.

Figure 1 presents the XRF spectra of CuO/PANI nanocomposites with increasing CuO nanoparticle concentrations (7 wt%, 14 wt%, and 21 wt%). Within this energy window, the prominent peaks are attributed to the characteristic X-ray emissions of copper. The copper peaks significantly intensified upon increasing the CuO content from 7 wt% to 14 wt% but exhibited negligible change between 14 wt% and 21 wt%. This behavior likely results from nanoparticle agglomeration, indicating saturation in nanoparticle dispersion and limited incremental addition of accessible copper sites at higher loading [35]. This trend indicates a higher copper content within the nanocomposites, as expected. The consistent presence and growth of these peaks across the samples confirm the incorporation of CuO NPs into the PANI matrix.

### 3.2. Optical Profilometer

**Figure 2:**
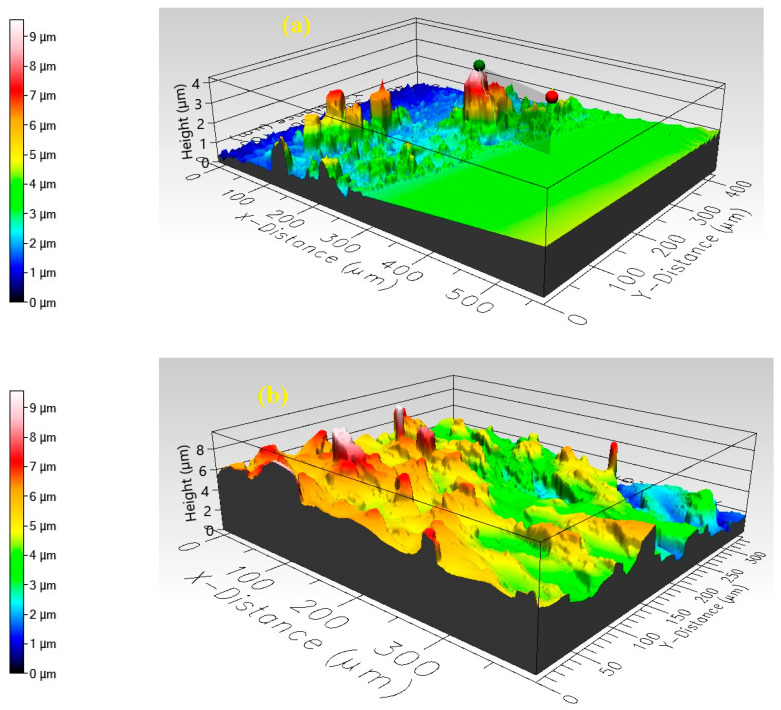
3D surface topography images of (**a**) PANI and (**b**) CuO/PANI nanocomposites obtained using a Filmetrics Profilm 3D optical profilometer.

Figure 2 illustrates the three-dimensional surface profiles of pristine PANI and PANI nanocomposites embedded with copper oxide CuO NPs, captured via a Filmetrics Profilm 3D optical profilometer. These visualizations offer detailed information on the films’ surface structures and roughness. The topography of pure PANI (Figure 2a) is relatively smooth, displaying minor undulations and small-scale features. Color variations in the image indicate differences in surface elevation, with distinct colors representing various height levels. In contrast, the CuO/PANI nanocomposite surface (Figure 2b) exhibits increased roughness, appearing more textured with numerous peaks and valleys. This enhanced texture is likely due to the incorporation of CuO NPs into the PANI, which disrupts the film’s smoothness, resulting in surface protrusions and depressions. Although exact film thicknesses cannot be directly ascertained from these two-dimensional images, the three-dimensional profilometer data allow for estimation. By analyzing vertical height variations across the surface, one can determine the average film thickness and assess its uniformity throughout the sample.

### 3.3. FTIR Spectra

**Figure 3:**
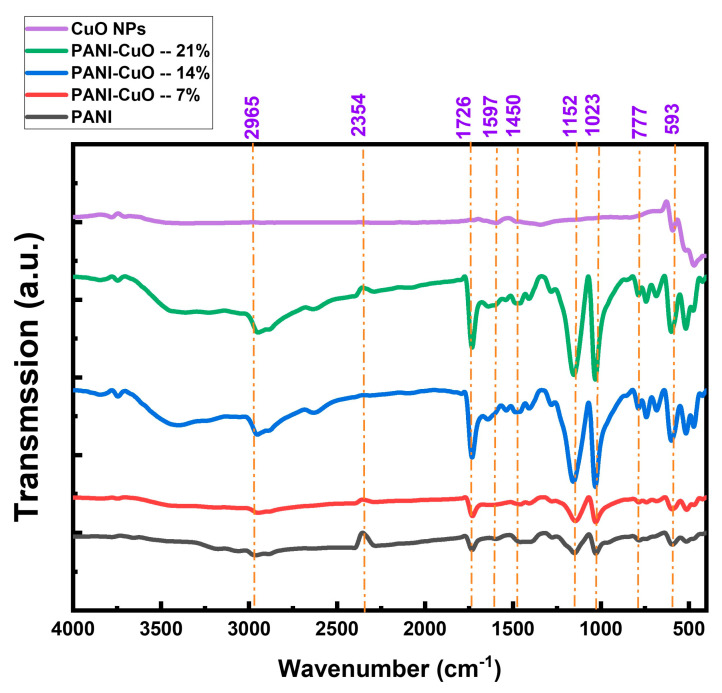
FTIR spectra of PANI and CuO/PANI nanocomposites.

Figure 3 illustrates the FTIR spectra of pristine CuO NPs, PANI, and its nanocomposites with copper oxide CuO NPs at loadings of 7%, 14%, and 21 wt%. These spectra elucidate the molecular structures and bonding interactions within the materials. A prominent absorption band at 2965 cm^−1^ corresponds to carbon–hydrogen (C–H) vibrational modes [36,37,38,39]. The absorption at 2354 cm^−1^ is linked to N–H stretching and unsaturated amine groups [37]. The peak at 1726 cm^−1^ is indicative of carbonyl (C=O) stretching vibrations, confirming the presence of CSA as a dopant [38,39,40,41].

Minor peaks near 1597 cm^−1^ and 1450 cm^−1^ are attributed to C=N stretching in the quinoid and benzenoid segments of the PANI chain, respectively [42,43]. The band around 1271 cm^−1^ is associated with C–N stretching in the benzenoid rings. Additional peaks at approximately 1152 cm^−1^ and 777 cm^−1^ correspond to C–H in-plane and out-of-plane bending vibrations, respectively [44]. The absorption at 593 cm^−1^ is ascribed to S–O stretching in sulfonated groups attached to aromatic rings [45], while the peak at 504 cm^−1^ is linked to C–S stretching vibrations [46]. Several notable observations can be made from the spectral features. Firstly, the observed increase in peak intensities upon the incorporation of CuO NPs can be attributed to the following factors. The addition of CuO NPs introduces strong interactions between the PANI matrix and CuO, including hydrogen bonding and electrostatic interactions. These interactions reinforce vibrational modes, leading to increased absorption intensities. Additionally, CuO NPs influence the local electronic environment of PANI, modifying the dipole moments of functional groups. This enhances IR absorption due to higher polarity and charge redistribution. Furthermore, CuO NPs may facilitate protonation effects within PANI, leading to enhanced doping levels, which alter vibrational modes, particularly in the C=N (quinoid) and C–N (benzenoid) regions. Secondly, the slight peak shifts observed in C=N (1597 cm^−1^), C–N (1271 cm^−1^), and S–O (593 cm^−1^) indicate strong CuO–PANI interactions, confirming successful nanocomposite formation. In addition, the broadening of peaks, particularly in the quinoid (1597 cm^−1^) and benzenoid (1450 cm^−1^) regions, suggests an increase in delocalized charge carriers, enhancing conductivity. Thirdly, the distinct absorption peaks at lower wavenumbers (~500–600 cm^−1^) correspond to Cu–O stretching vibrations, verifying the presence of CuO in the PANI matrix.

**Figure A2:**
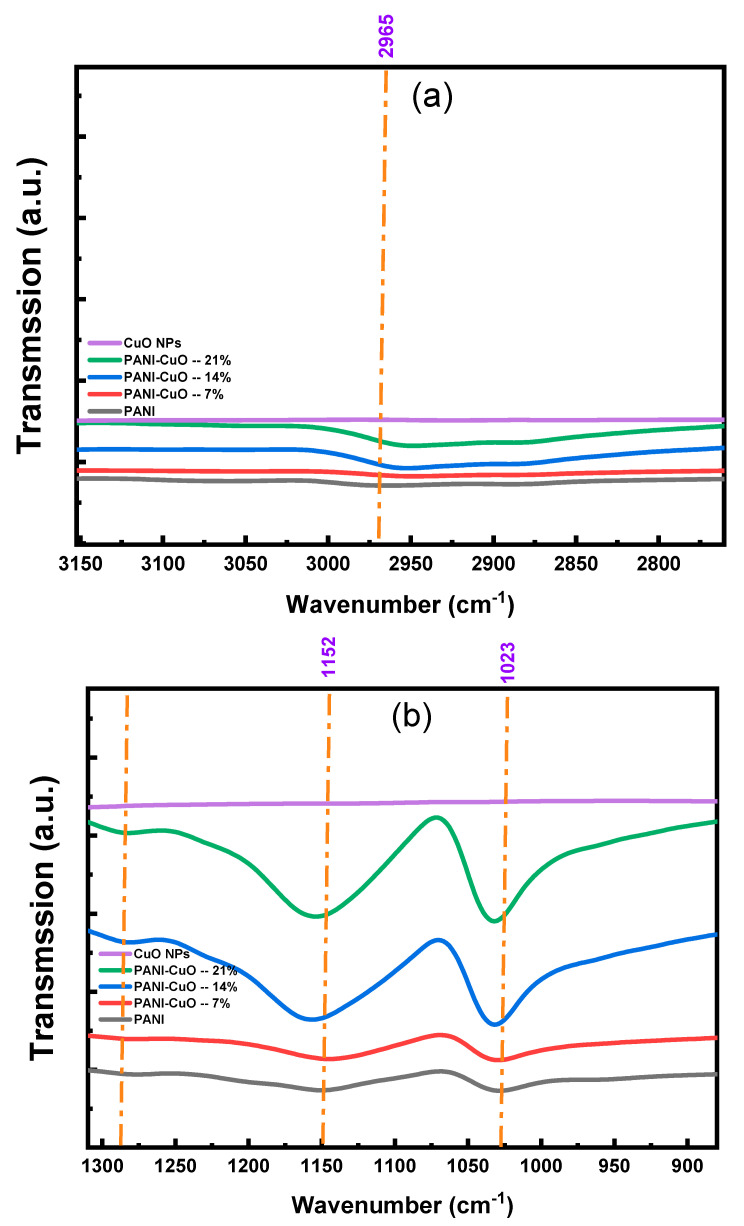

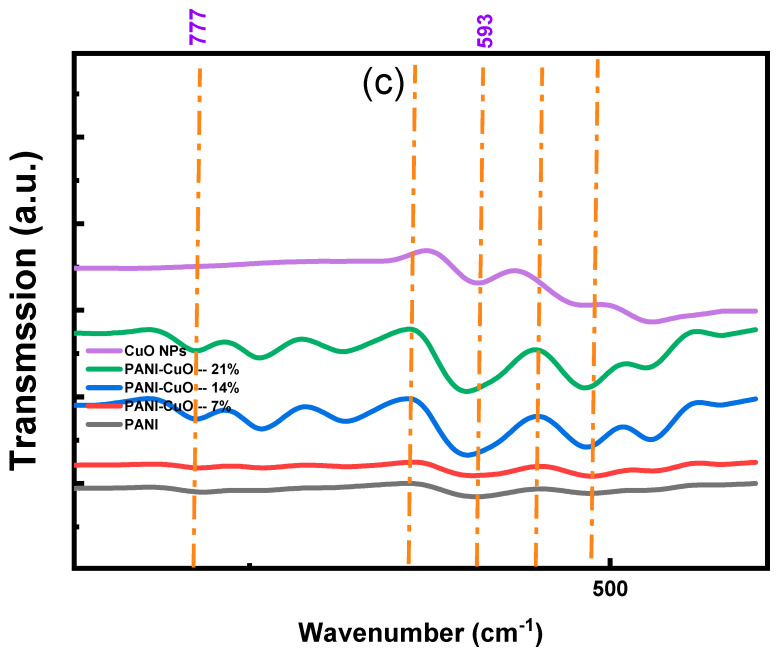
FTIR spectra of PANI and CuO/PANI nanocomposites. (**a**) 3000–2800 cm^−1^ region (aromatic C–H stretch): a vertical dashed line at 2965 cm^−1^ marks the C–H stretching mode in pure PANI. Overlaid arrows show its progressive red shift to 2958 cm^−1^ (7 wt% CuO), 2955 cm^−1^ (14 wt%), and 2952 cm^−1^ (21 wt%). (**b**) 1650–1000 cm^−1^ region (quinoid/benzenoid C=N and C=C stretches; C–N stretch): dashed lines at 1597 cm^−1^ (quinoid C=N) and 1495 cm^−1^ (benzenoid C=C) denote the pure PANI band positions. In the composites, the quinoid mode shifts to 1605→1601→1598 cm^−1^ and the benzenoid mode to 1503→1499→1496 cm^−1^ for 7, 14, and 21 wt%, respectively. The C–N stretching band at 1308 cm^−1^ likewise moves to 1315→1310→1306 cm^−1^ across the same loadings. (**c**) 800–500 cm^−1^ region (out-of-plane ring vibrations and Cu–O lattice modes): reference lines at 777 cm^−1^ and 593 cm^−1^ indicate pure PANI in plane-ring modes. In the 21 wt% composite, these features shift to 781 cm^−1^ and 597 cm^−1^, respectively, evidencing polymer–oxide interactions. Additionally, the Cu–O lattice vibration near 450 cm^−1^ grows in intensity with increasing CuO content, confirming the higher nanoparticle concentration in the films.

As shown in Figure A2a–c, the characteristic PANI bands at 2965, 1597, 1495, 1308, 1152, 1023, 777, and 593 cm^−1^ undergo systematic shifts upon CuO incorporation. For example, the quinoid C=N stretch moves from 1597→1605 cm^−1^ at 7 wt%, gradually returning toward the original position at higher loadings, indicating a strong, but tunable, polymer–oxide interaction.

### 3.4. UV-Vis-NIR Analysis

**Figure 4:**
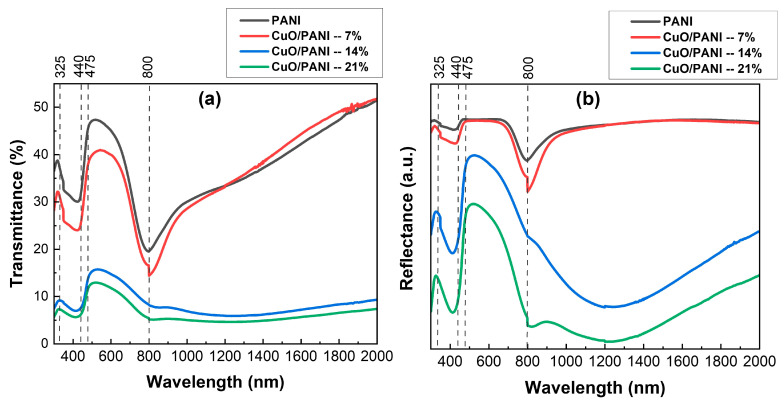
(**a**) Transmittance and (**b**) reflectance spectra of the PANI and CuO/PANI nanocomposites.

**Figure 5:**
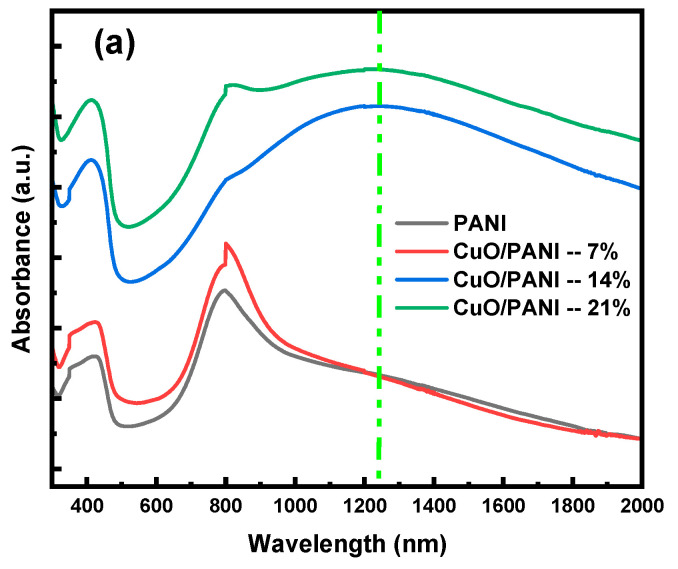

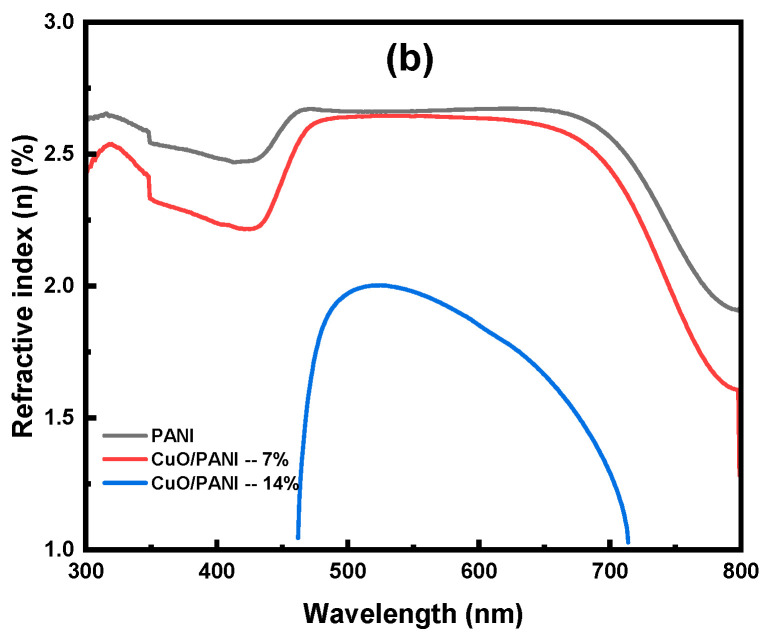
(**a**) Absorbance and (**b**) the refractive index (n) of the PANI and CuO/PANI nanocomposite films.

The UV-Vis spectroscopy technique was employed to examine the optical characteristics of the synthesized samples. Figure 4a,b illustrate the transmittance and reflectance spectra. The PANI and their nanocomposites generally showed a trend of reduced transmittance across the examined wavelength range. However, pure PANI films exhibited higher transmittance compared to the CuO/PANI nanocomposite films. The transmittance of pure PANI showed a slight increase from around 36% to 39% between 300 nm and 325 nm, followed by a further rise to 47% between 440 nm and 475 nm. After this range, transmittance steadily decreased until reaching 800 nm, after which it increased again to 53% in the near-infrared (NIR) region. As depicted in Figure 5a, the addition of increasing concentrations of CuO NPs into the PANI resulted in a noticeable reduction in transmittance across the visible spectrum.

This decline can be attributed to the enhanced light absorption within the nanocomposite material. The observed absorption is likely due to electronic transitions within the PANI matrix, potentially involving bandgap states that are influenced by the inclusion of CuO NPs. The absorption edge, which marks the beginning of electronic transitions, is found in the high-energy region, with an estimated energy exceeding 3.5 eV, aligning with findings from earlier studies [47]. Figure 5b shows a clear reduction in reflectance as the content of CuO NPs in the PANI increases. This effect can be attributed to several interconnected factors: CuO NPs inherently absorb light over a broad range of wavelengths, and when incorporated into the PANI, they enhance the absorption of incident light, leading to a decrease in reflectance. Additionally, the integration of CuO NPs with the PANI results in the formation of interfaces between the polymer and the nanoparticles, further influencing the material’s optical properties. The interfaces within the composite can cause the scattering of incident light, causing it to deviate in different directions. This scattering effect diminishes the light reflected directly from the surface, thereby lowering the overall reflectance. Additionally, the inclusion of CuO NPs modifies the refractive index of the composite, which can disrupt the optical impedance matching at the material’s surface, further contributing to the reduction in reflectance [48,49,50].

Figure 5a illustrates the absorbance spectra of the synthesized films, demonstrating that CuO/PANI nanocomposites exhibit superior absorbance compared to pure PANI. This enhancement can be attributed to multiple factors. Firstly, CuO NPs inherently absorb light, with their absorption characteristics influenced by factors such as particle size, morphology, and crystalline structure. These attributes promote electronic-band transitions, thereby contributing to the increased absorbance observed in the nanocomposites [51]. Secondly, the integration of CuO NPs within the PANI matrix introduces a more complex light-propagation pathway. This extended optical path enhances the interaction between incident photons and PANI chromophores, increasing the likelihood of photon absorption and ultimately leading to a higher overall absorbance in the nanocomposites [52,53]. As depicted in Figure 4a, the absorbance spectra of the synthesized films exhibit three well-defined absorption peaks at distinct wavelengths. The pristine PANI film demonstrates three characteristic peaks; the first peak at 350 nm corresponds to the π-π* electron transitions within the benzenoid segments of the PANI backbone, indicative of the intrinsic electronic structure of the polymer. Secondly, a broader absorption feature appears around 430 nm, attributed to polaron-π* transitions, signifying the formation of polaronic states within the polymer matrix. Thirdly, a peak at 795 nm is linked to π-polaron transitions, representing interactions between the π-electron system and charge carriers in the polymer. A notable broad absorption band emerges at 1242 nm in films containing 14 wt% and 21 wt% CuO NPs, exhibiting a redshift. This spectral shift suggests a reduction in the optical-band gap, which can be attributed to strong interfacial interactions between CuO NPs and the PANI. These interactions likely facilitate charge delocalization and enhance electronic coupling within the nanocomposite system [48].

Figure 5b presents the refractive index (*n*) spectra for both pristine PANI and CuO/PANI nanocomposites. The refractive index is a critical parameter that influences the optical behavior of these materials, affecting their interaction with light across different wavelengths.

The complex refractive index, expressed as (N = *n* + i*k*), provides a thorough description of a material’s optical behavior. In this notation, *n* signifies the real component, which determines the phase velocity of light as it propagates through the medium. Meanwhile, *k*, known as the extinction coefficient, quantifies the attenuation of the electromagnetic wave, arising from both absorption and scattering effects. This parameter is essential for understanding light–matter interactions and is widely utilized in optical-material characterization [49]. This advanced framework enables a comprehensive examination of light–matter interactions in thin films, capturing both the velocity of light propagation and the associated attenuation effects. (*n*) of the samples was calculated using Equation (1), which integrates reflectance (*R*) and the extinction coefficient (*k*). This methodology provides an accurate assessment of the material’s optical behavior, effectively considering its intrinsic absorption properties.

(1) $$n=\left(\frac{1+R}{1-R}\right)+\sqrt{\frac{4R}{{\left(1-R\right)}^{2}}-K^{2}}$$

**Figure 6:**
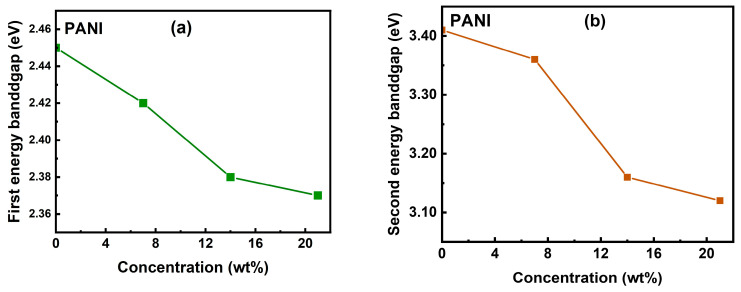
Variation of the first optical bandgap (**a**) and the second optical bandgap (**b**) for the PANI and CuO/PANI nanocomposites.

The precise characterization of (*n*) of thin films is crucial for the design and optimization of photonic devices. Figure 6b presents the dispersion of (*n*) as a function of wavelength for the synthesized films. Understanding this dispersion behavior is essential, as it directly influences the optical performance of components such as waveguides, coatings, and modulators. Accurate refractive index data enable the prediction of light-propagation characteristics, interference effects, and overall device efficiency, thereby guiding the engineering of materials and structures in advanced optical applications [52,53]. The refractive index (*n*) of PANI varies between 1.8 and 2.6 across the 300 to 800 nm wavelength range. CuO/PANI nanocomposites exhibit refractive indices ranging from 1.0 to 2.6 across the same spectral range. The reduction in refractive index compared to PANI films is primarily due to the incorporation of CuO NPs, which inherently have a lower refractive index than PANI. This results in a dilution effect that lowers the overall refractive index of the composite. Additionally, both pristine PANI and CuO/PANI nanocomposites demonstrate a decreasing refractive index with increasing wavelength, a typical characteristic of normal dispersion observed in dielectric materials. The decrease in (*n*) of CuO/PANI nanocomposites relative to pure PANI can be ascribed to several contributing factors. These factors may include the incorporation of CuO NPs, which influence the overall electronic structure and optical properties of the composite material. Additionally, interactions between CuO and PANI could lead to changes in charge distribution, electron mobility, or the formation of hybridized interfaces that alter the material’s optical behavior. The CuO/PANI nanocomposite containing 14% CuO exhibits a broad peak in its refractive-index spectrum. As the CuO nanoparticle concentration increases, the intensity of this peak decreases. This behavior can be ascribed to the increasing concentration of CuO NPs, which disrupts the polymer matrix of PANI. This disruption leads to a change in the electronic polarizability of the composite, resulting in a reduced refractive index. These findings highlight the intricate relationship between the incorporation of nanoparticles and the resulting optical properties of polymer nanocomposites [11].

**Figure A3:**
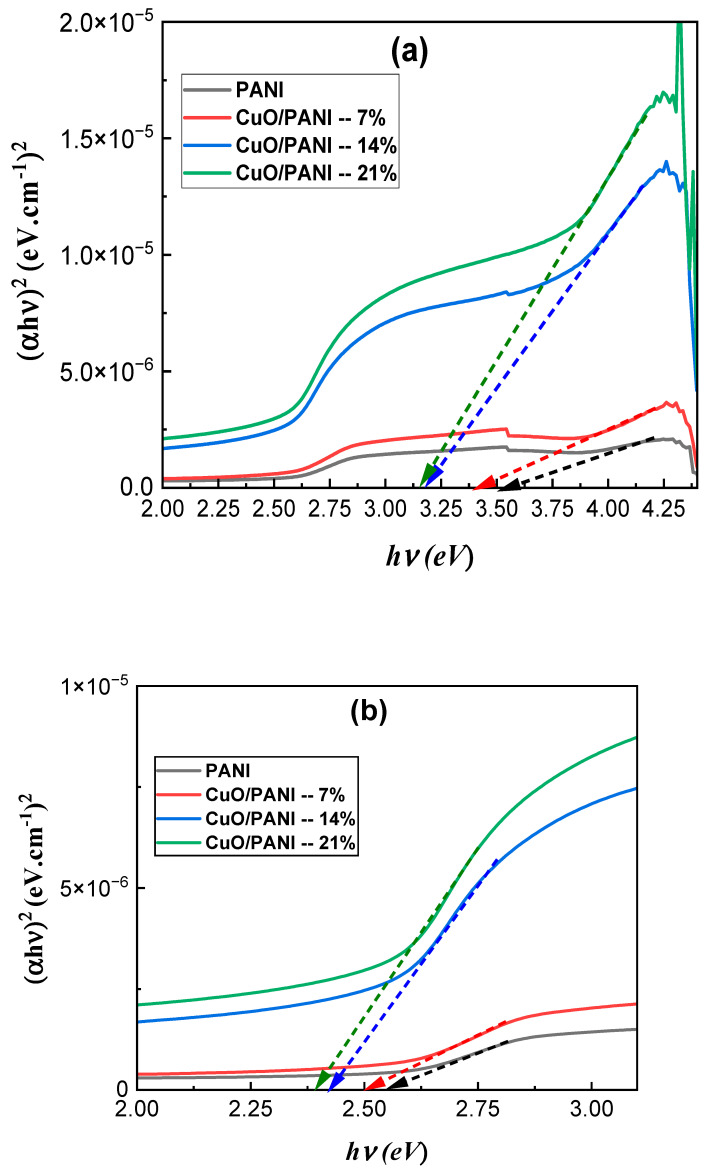
Variation of (*αhυ*)^2^ versus the incident photon energy for PANI and CuO/PANI nanocomposites showing the behavior of interband transitions (**a**) benzenoid rings (B) (S_2_) and (**b**) quinoid rings (Q) (S_1_).

The optical bandgap energy (*Eg*) for all samples was determined using the Tauc plot method, as outlined in reference [54]. The plot was constructed by plotting (αhν)2 on the vertical axis against photon energy (hν) on the horizontal axis, revealing two distinct linear regions. This analysis identified two optical bandgaps in the PANI film, with values of *Eg*1 = 2.45 eV and *Eg*2 = 3.41 eV. The lower energy bandgap (*Eg*1) is associated with polaron transitions occurring within the quinoid (Q) rings of the PANI structure. The higher energy bandgap (*Eg*2) is linked to the π-π* electronic transitions within the benzenoid (B) rings [55,56,57,58]. The relatively small bandgap energy (*Eg*) of PANI can be ascribed to the increased density of conjugated PANI chains, as the bandgap energy is inversely related to the degree of conjugation within the polymer [59]. In CuO/PANI nanocomposites, the primary bandgap energy (*Eg*1) was observed to be 2.42 eV at 7 wt.%, 2.38 eV at 14 wt.%, and 2.37 eV at 21 wt.%, while the secondary bandgap energy (*Eg*2) values were 3.36 eV at 7 wt.%, 3.16 eV at 14 wt.%, and 3.12 eV at 21 wt.% as summarized to Table 1. The reduction in both *Eg*1 and *Eg*2 relative to pure PANI is likely due to the incorporation of CuO, which alters the electronic structure of the nanocomposite. This interaction may lead to enhanced charge-transfer properties, influencing the overall bandgap energies and reflecting changes in the material’s conjugation and electronic characteristics [60,61]. Figure A3 illustrates the variation of (αhυ)2 versus the incident photon energy for PANI and CuO/PANI nanocomposites. Notably, CuO NPs exhibit two distinct band gaps due to their unique electronic structure and quantum-size effects. Typically, these band gaps are classified as follows. Primary Band Gap (*Eg*1) corresponds to the intrinsic bandgap of CuO, which generally falls within the range of 1.2–2.5 eV, depending on factors like particle size, synthesis method, and structural defects. Secondary Band Gap (*Eg*2) refers to a higher energy transition often observed in nanostructured CuO, typically in the range of 3.0–3.5 eV. This secondary bandgap arises due to interband transitions, defect states, or the influence of nanostructuring, which alters the material’s electronic properties [61,62]. Figure 6a,b illustrate the dependence of gaps *Eg*1 and *Eg*2 on CuO concentration in the CuO/PANI nanocomposites, in comparison to pure PANI. The observed decline in the optical band gap with increasing CuO NP content indicates enhanced charge carrier delocalization and improved electronic interactions at the CuO–PANI interface, leading to a more efficient charge-transfer mechanism. The incorporation of nanoparticles (NPs) into nanocomposites plays a pivotal role in modifying their electronic structure, facilitating the formation of new electronic states. The bandgap of the nanocomposite acts as a key interface between the polymer’s highest occupied molecular orbital (HOMO) and the conduction states of the embedded nanoparticles. This energy alignment is fundamental to charge transport processes and is influenced by factors such as nanoparticle dispersion and polymer-NP interfacial interactions within the composite matrix. Additionally, structural characteristics, including phase separation and aggregation behavior, further dictate the electronic properties of the material.

### 3.5. Electrical Conductivity

The DC electrical conductivity (σ) of the film was evaluated utilizing the four-point probe technique, a well-established method known for its accuracy in measuring a material’s electrical transport properties. This technique effectively reduces contact resistance, allowing for precise resistivity determination and ensuring reliable conductivity assessments [63,64].

**Figure 7:**
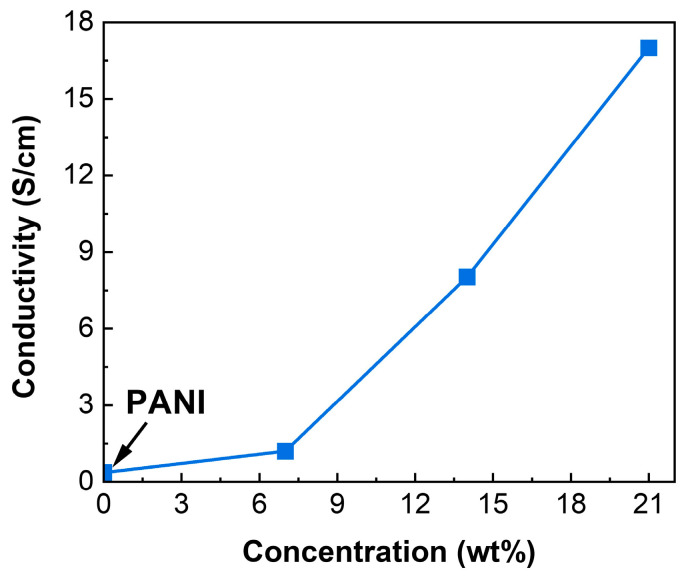
Electrical conductivity of the of PANI and CuO/PANI nanocomposites.

Figure 7 illustrates (σ) trends for both PANI and CuO/PANI nanocomposites.The pristine PANI demonstrates a conductivity of 0.36 S/cm (see Table 2), which is relatively high due to the formation of an extensive network of conjugated polymer chains during the polymerization process, a phenomenon extensively reported in the literature [63,64,65,66,67]. Upon the incorporation of CuO NPs, the electrical conductivity of CuO/PANI nanocomposites exhibits a progressive increase, reaching a maximum of 17.00 S/cm at 21 wt.% CuO loading. This significant enhancement in conductivity can be primarily ascribed to the inherently low optical bandgap of CuO nanoparticles, typically ranging between 1.2 eV and 2.5 eV, which facilitates charge-carrier excitation and transport [68].

### 3.6. Surface Morphology

**Figure 8:**
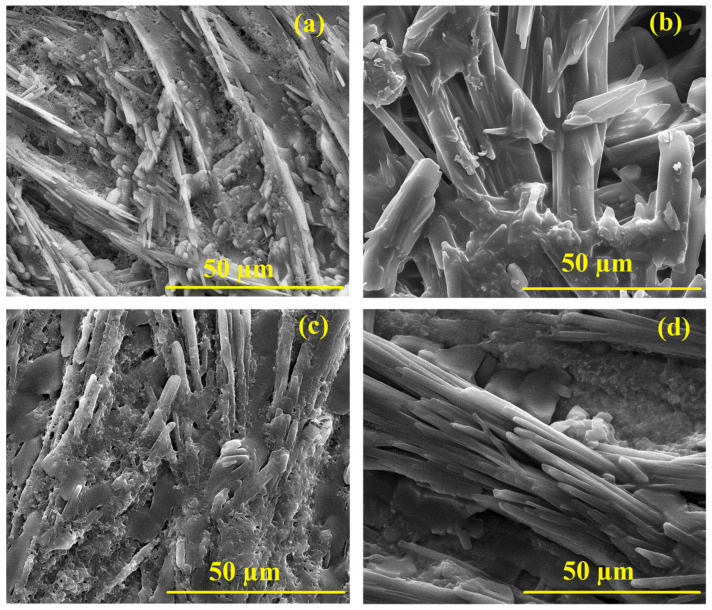
SEM images of the samples at various CuO NPs: (**a**) PANI (without NPs), (**b**) 7 wt% CuO NPs, (**c**) 14 wt% CuO NPs, and (**d**) 21 wt% CuO NPs. (all at 3000×). Higher-magnification (at 400,000×). Views showing individual CuO NPs are provided in Figure A5.

Figure 8 shows the SEM images of the PANI at varying CuO NPs contents, offering valuable insights into the morphology of the nanocomposites with increasing CuO NP concentration. The images present the following observations. The Pure PANI morphology is scientifically described as having relatively uniform polymeric domains interspersed with minor irregularities and subtle fibrillar-like structures rather than homogeneous smoothness. Upon incorporating 7 wt% CuO NPs, the SEM image reveals a slight increase in surface roughness, with nanoparticles becoming visible as small, well-dispersed agglomerates on the surface of the PANI.

**Figure A5:**
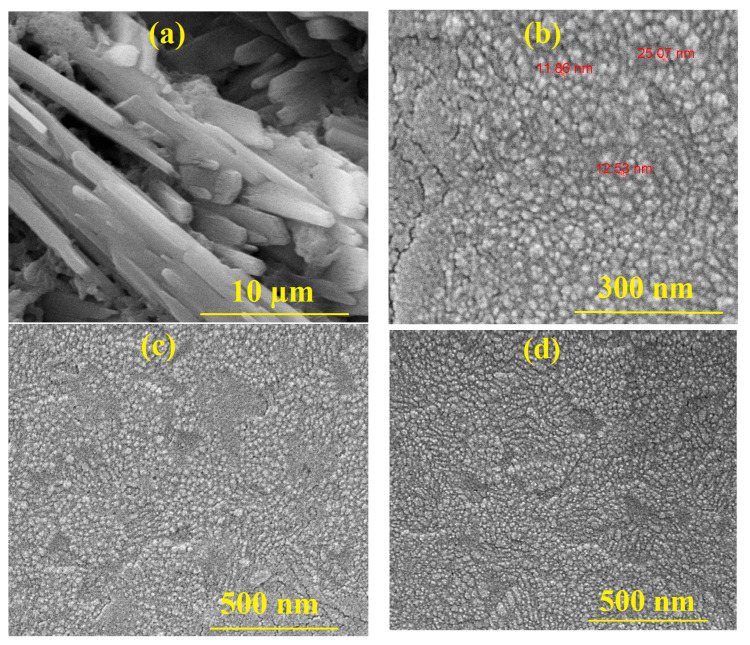
Higher-magnification (at 400,000×) views showing individual CuO NPs.

These CuO NPs appear to be interacting with the PANI chains, likely leading to some degree of interface modification between the polymer and nanoparticles. At this concentration, CuO NPs seem to enhance the surface texture of the PANI film without causing significant clustering or aggregation. At 14 wt% CuO NPs, the film exhibits larger agglomerates of CuO NPs, and the surface morphology becomes more heterogeneous. The presence of CuO NPs seems to induce further roughening of the surface, as evidenced by the larger nanoparticle clusters and their more irregular distribution across the film. This increased aggregation may indicate that the balance between polymer–nanoparticle interaction and nanoparticle dispersion is becoming less optimal, potentially limiting the uniformity of the nanocomposite at this concentration. At the highest loading of 21 wt% CuO NPs, the SEM image shows significant aggregation and clustering of the CuO NPs, which likely results in poor dispersion within the PANI matrix. The surface is highly rough and heterogeneous, with CuO NPs forming larger clusters that might disrupt the polymer’s overall structural integrity. This suggests that, beyond a certain concentration, the ability of the polymer matrix to maintain a uniform distribution of nanoparticles is overwhelmed, leading to a decrease in the homogeneity and potential degradation in the overall performance of the nanocomposite. While low-magnification SEM (Figure 8), confirms overall film homogeneity, high-magnification images (Figure A5) reveal well-dispersed CuO NPs of an ~11–25 nm diameter in all composites, with no evidence of aggregation up to 21 wt% loading.

### 3.7. X-Ray Diffraction

The XRD analysis of PANI reveals distinct structural characteristics between its powder and film forms. PANI typically displays broad diffraction peaks in its powdered state, indicating a semi-crystalline structure with crystalline and amorphous regions. This broadness suggests limited long-range molecular order [69,70]. Conversely, PANI films often exhibit sharper and more defined diffraction peaks in their XRD patterns, signifying enhanced crystallinity and improved molecular ordering upon film formation. For instance, studies have reported distinct peaks at specific 2θ angles, corresponding to particular crystal planes, which are more pronounced in the film form than the powder [71]. These observations underscore the influence of processing methods on the structural organization of PANI, with film formation promoting a higher degree of crystallinity compared to its powdered counterpart.

**Figure 9:**
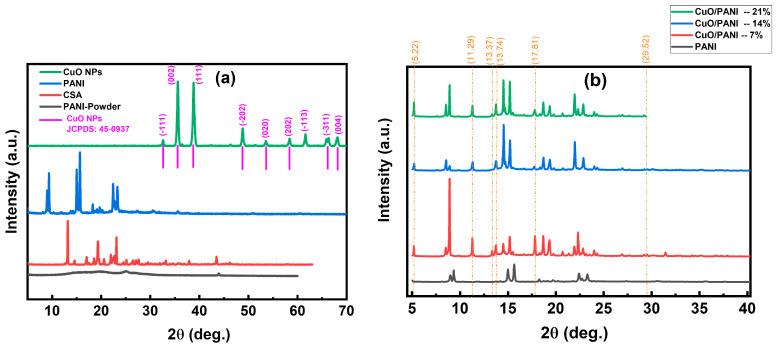
(**a**) XRD analysis of PANI powder, CSA, PANI film, and CuO NPs and (**b**) X-ray diffraction analysis of PANI and CuO/PANI nanocomposites.

The XRD patterns of CuO/PANI nanocomposites reveal the emergence of additional diffraction peaks at 2θ = 5.22°, 11.29°, 13.37°, 13.74°, 17.81°, and 29.52°. These peaks are consistently observed across samples containing 7 wt.%, 14 wt.%, and 21 wt.% CuO NPs, as illustrated in Figure 9. The presence of CuO NPs leads to an increased number of diffractions features within the PANI matrix, suggesting alterations in its crystallographic arrangement, likely due to interactions between CuO and PANI at the molecular level. The presence of additional diffraction peaks in the XRD pattern signifies the incorporation of CuO NPs into the PANI. This occurrence is primarily driven by several structural factors. Firstly, CuO NPs exhibit a well-defined crystalline nature, and their integration with PANI results in unique phase interactions that manifest as new diffraction signals. Secondly, the distinct crystallographic features of CuO contribute to sharp diffraction peaks, highlighting the structural stability of the nanocomposite. Moreover, the emergence of these peaks provides strong evidence of intermolecular interactions between PANI and CuO, which may induce the formation of novel crystalline arrangements or intricate phase transformations within the composite material [72].

All synthesized nanocomposites exhibited diffraction patterns like those of pristine PANI films. However, a systematic shift of the diffraction peaks toward lower 2θ values was observed, along with noticeable variations in peak intensity. This shift suggests an increase in interchain spacing within the polymer matrix due to interactions between CuO NPs and the PANI backbone, leading to structural modifications. XRD analysis further indicates that the diffraction peak intensities of CuO/PANI nanocomposites are significantly higher compared to PANI. This enhancement can be ascribed to multiple factors. The incorporation of CuO NPs enhances the overall crystallinity of the composite by acting as nucleation centers, promoting a more ordered molecular arrangement. This structural organization reduces amorphous content and reinforces periodicity within the polymer lattice, resulting in sharper and more intense diffraction peaks. Increased crystallinity typically correlates with enhanced diffraction intensity, as it improves constructive interference during XRD analysis. In addition, the influence of nanoparticle size is a key factor in determining diffraction behavior. As the nanoparticle dimensions decrease, the number of scattering centers increases, resulting in broader, yet more pronounced, diffraction peaks. This phenomenon is ascribed to the higher surface-to-volume ratio, which amplifies diffraction intensity due to enhanced structural interactions. The observed diffraction patterns indicate a significant degree of integration between CuO NPs and the PANI matrix, suggesting a well-structured nanocomposite with improved crystallinity and modified lattice characteristics. Table 3 presents the calculated average crystallite size (D) of CuO NPs, calculated using the Scherrer formula, Equation (2):

(2) $$D=\frac{K\lambda }{\beta (2\theta )cos(\theta )}$$

where *k* is the shape factor (typically 0.94), λ represents the X-ray wavelength, β(2θ) is the full width at half maximum (FWHM) of the diffraction peak, and θ is the Bragg diffraction angle. Additionally, the lattice strain (ε) and dislocation density (β)were evaluated using the following expressions, Equations (3) and (4):

(3) $$\varepsilon =\frac{\beta cos(\theta )}{4}$$

(4) $$\delta =\frac{1}{D^{2}}$$

XRD analysis determined that CuO NPs had an average crystallite size of around 80 nm. As the CuO NPs content increased, a corresponding growth in crystallite size was noted. This trend may be ascribed to the role of CuO NPs in modifying the surrounding matrix, likely affecting nucleation dynamics and growth mechanisms. Such interactions could promote enhanced particle coalescence or altered energy barriers during crystallization, ultimately influencing the structural evolution of the material [72].

While prior studies have explored the general synthesis and characterization of PANI–CuO composites [11,12], the present work introduces a novel approach by optimizing CuO nanoparticle concentrations (7, 14, and 21 wt%) via electrochemical deposition directly onto ITO substrates. This technique systematically evaluated concentration-dependent structural, optical, morphological, and electrical properties. Notably, the study demonstrates a consistent reduction in optical bandgap energies, correlated with extended conjugation in polymer chains, alongside quantifiable increases in crystallite size and shifts in strain and dislocation density. FTIR analysis reveals new insights into CuO–PANI interactions at the molecular level, while the electrical conductivity of the nanocomposites shows a significant enhancement, reaching 17.0 S/cm at higher CuO loadings. These results collectively represent a substantial advancement beyond the existing literature, both in methodology and in the depth of characterization.

## 4. Conclusions

In summary, this research effectively developed nanocomposite films by embedding CuO NPs in varying weight proportions within a PANI, a conductive polymer, through a refined electrochemical deposition method, applied directly to ITO. The protonated PANI leads to improvements in both the structural integrity and optical characteristics of the final nanocomposite material. In our study, we employed a range of analytical techniques to thoroughly assess the properties of the nanocomposite films. UV-Vis spectroscopy was used to examine their optical behavior, while XRD and FTIR provided insights into the atomic structure and chemical bonding. SEM enabled the observation of morphological features. The UV-Vis analysis revealed significant light absorption by PANI at specific wavelengths. Additionally, the FTIR spectra of CuO/PANI showed notable peak shifts and broadening, suggesting potential interactions between the polymer matrix and the CuO NPs. The films exhibited a range of refractive indices, emphasizing their suitability for optical applications. Moreover, a reduction in the optical bandgap energy was noted in CuO/PANI nanocomposites, alongside an increase in electrical conductivity as the CuO NPs concentration was elevated. This trend can be ascribed to the integration of CuO NPs, which enhanced the conjugation of π-bonds within the PANI matrix and caused disruption in the polymer chain’s regular structure. The SEM examination of the pristine PANI demonstrated predominantly smooth and uniform surface morphology. In contrast, XRD results indicated a subtle shift in the diffraction peaks of the CuO/PANI nanocomposites, suggesting possible structural alterations arising from the formation of the nanocomposite. Although polyaniline-based materials are inherently electrochromic, and the CuO/PANI nanocomposites reported here exhibit pronounced color differences under different redox conditions, we have not yet performed quantitative switching-cycle or coloration-efficiency measurements.

## Figures and Tables

**Table 1:** Optical bandgaps of the PANI and CuO/PANI nanocomposites.

Sample	Optical Bandgap 1 (*Eg*1) (eV)	Optical Bandgap 2 (*Eg*2) (eV)
PANI	2.45	3.41
CuO/PANI—7 wt.%	2.42	3.36
CuO/PANI—14 wt.%	2.38	3.16
CuO/PANI—21 wt.%	2.37	3.12

**Table 2:** Summarize the conductivity of PANI and CuO/PANI nanocomposites as a function of NPs content.

Sample	Conductivity σ [S/cm]
PANI	0.36
CuO/PANI—7 wt.%	1.20
CuO/PANI—14 wt.%	8.02
CuO/PANI—21 wt.%	17.00

**Table 3:** Crystallographic properties of PANI and CuO/PANI nanocomposites.

Sample	Crystalline Grain Size (D) (nm) at (2θ = 15.2 °)	Strain (*ε*) (× 10^−4^)	Dislocation Density (*δ*) (× 10^13^ m^−2^)
PANI	80	4.55	15.8
CuO/PANI—7 wt.%	108	3.37	8.65
CuO/PANI—14 wt.%	99	3.66	1.02
CuO/PANI—21 wt.%	121	2.99	6.79

**Figure A4:**
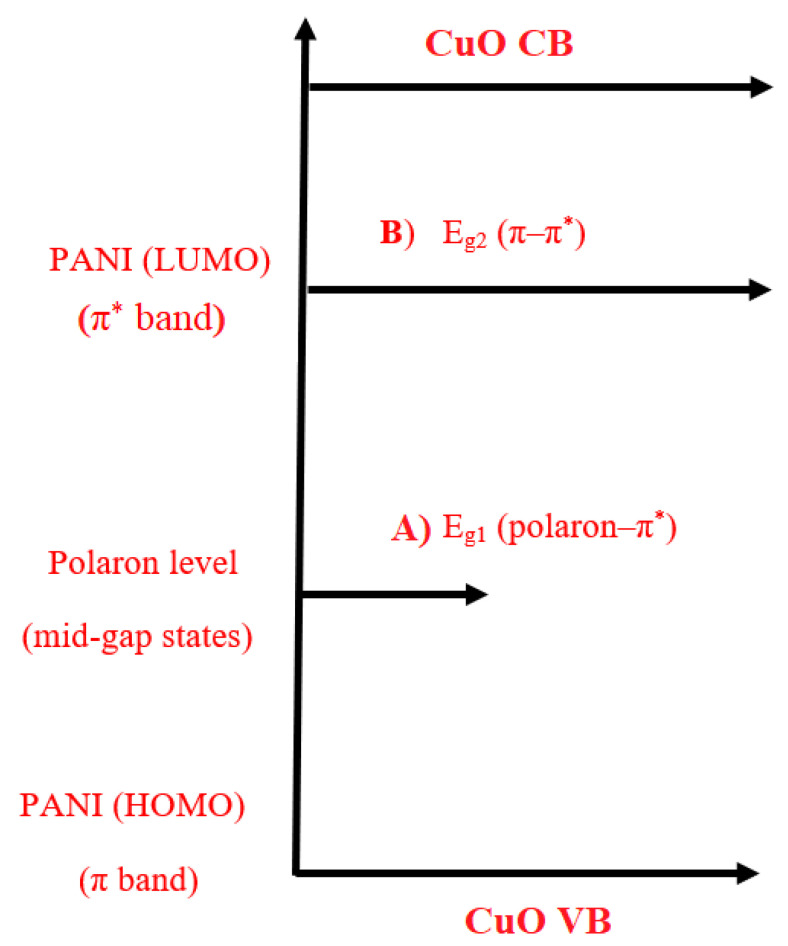
Energy-level schematic, *Eg*1 (Bandgap 1): transition from the lower polaron state to the PANI–π* band, *Eg*2 (Bandgap 2): full π–π* excitation across the benzenoid ring. CuO transitions are shown overlapping, with their own intrinsic gap and higher-energy defect/interband transitions.

## Data Availability

The original contributions presented in this study are included in the article. Further inquiries can be directed to the corresponding author.

## Appendix A. Supporting Information and Data

### Appendix A.2. FTIR Spectra

Figure A2: Annotated FTIR peak-shift analysis for PANI–CuO nanocomposites.

### Appendix A.3. Optical Bandgap Energy

The bandgap energies (*E_g_*) of the samples were estimated using Tauc plots, where a linear extrapolation was performed to the point at which (*αhν*)^2^ intersects with the energy axis (*hν*) at zero.

The Tauc plots exhibited two distinct linear regions for all the nanocomposite films, indicating the presence of multiple optical transitions. The first bandgap energy (S3), ranging from 2.37 to 2.42 eV, is likely associated with the initial stages of light absorption, leading to the formation of bound electron-hole pairs or excitonic states. This observation is consistent with previous studies [66]. Additionally, a second set of bandgap energies, ranging from 3.36 to 3.12 eV, was identified, which corresponds to the energy difference between the highest occupied molecular orbital (HOMO) and the lowest unoccupied molecular orbital (LUMO) of the material.

### Appendix A.4. Optical Bandgaps of the PANI and CuO/PANI Nanocomposites

Figure A3 presents a new energy-level diagram illustrating the PANI valence band (HOMO) and conduction band (LUMO), as well as the polaron levels introduced by doping and dedoping within the band gap. Arrow A denotes the lower-energy polaron transition (*Eg*1), while Arrow B represents the higher-energy π–π* transition (*Eg*2). For the CuO nanoparticles, the diagram also includes their bulk valence and conduction bands, highlighting how quantum-size effects and defect states can introduce a secondary transition (typically in the 3.0–3.5 eV range) that overlaps with PANI’s π–π* band and shifts depending on particle size. This figure supports our interpretation of the two linear regions observed in the Tauc plots: the lower-energy gap (*Eg*1) is attributed to polaron-related transitions within the doped PANI, and the higher-energy gap (*Eg*2) corresponds to the fundamental π–π* excitation of the benzenoid segments. In the CuO/PANI composites, the alignment between PANI’s mid-gap and π–π* levels and CuO’s conduction and valence bands accounts for the red-shifts and gap narrowing observed with increasing nanoparticle loading.

### Appendix A.5. Surface Morphology

Figure A5 now presents SEM micrographs of the 7 wt%, 14 wt%, and 21 wt% CuO/PANI films at 400,000× magnification. These high-resolution images resolve the individual CuO nanoparticles (average diameter ≈ 11–25 nm) embedded within the PANI matrix and illustrate their uniform dispersion.
